# A miniature solar device for overall water splitting consisting of series-connected spherical silicon solar cells

**DOI:** 10.1038/srep24633

**Published:** 2016-04-18

**Authors:** Yosuke Kageshima, Tatsuya Shinagawa, Takaaki Kuwata, Josuke Nakata, Tsutomu Minegishi, Kazuhiro Takanabe, Kazunari Domen

**Affiliations:** 1 Department of Chemical System Engineering, The University of Tokyo, 7-3-1 Hongo, Bunkyo-ku, Tokyo, 113-8656, Japan; 2 Division of Physical Science and Engineering, KAUST Catalysis Center (KCC), King Abdullah University of Science and Technology (KAUST), 4700 KAUST, Thuwal, 23955-6900, Saudi Arabia; 3 Kyosemi Corporation, 949-2 Ebisu-cho, Fushimi-ku, Kyoto, 612-8201, Japan

## Abstract

A novel “photovoltaics (PV) + electrolyzer” concept is presented using a simple, small, and completely stand-alone non-biased device for solar-driven overall water splitting. Three or four spherical-shaped p-n junction silicon balls were successfully connected in series, named “SPHELAR.” SPHELAR possessed small projected areas of 0.20 (3PVs) and 0.26 cm^2^ (4PVs) and exhibited working voltages sufficient for water electrolysis. Impacts of the configuration on the PV module performance were carefully analyzed, revealing that a drastic increase in the photocurrent (≈20%) was attained by the effective utilization of a reflective sheet. Separate investigations on the electrocatalyst performance showed that non-noble metal based materials with reasonably small sizes (<0.80 cm^2^) exhibited substantial currents at the PV working voltage. By combining the observations of the PV characteristics, light management and electrocatalyst performance, solar-driven overall water splitting was readily achieved, reaching solar-to-hydrogen efficiencies of 7.4% (3PVs) and 6.4% (4PVs).

The conversion of solar energy to electric power or chemical energy has been considered a key technology for overcoming energy and environmental issues and for establishing a clean and sustainable society. Because solar irradiance has daily, seasonal, and meteorological fluctuations and uneven regional distributions, the production of chemical energy carriers, such as hydrogen, from solar energy is an effective way to store energy for a practical and industrial large-scale operation^1^,^2^. As one of the candidates for solar-to-hydrogen (STH) generation, photoexcitation processes using semiconductors to drive water splitting redox reactions have attracted great attention^3^,^4^. One strategy is to use the electric power generated by photovoltaics (PVs) for water electrolysis, namely the “PV + electrolyzer” approach^5^,^6^,^7^,^8^,^9^,^10^. Conventional single junction solid-state PVs, such as Si, Cu(Ga,In)Se_2_, and CdTe, are designed to generate 0.6–0.9 V of an open-circuit voltage (*V*_OC_) utilizing semiconductors with band gaps of 1.0–1.5 eV^11^,^12^,^13^, while multi junction PVs generate greater *V*_OC_s than 1 V. Another group of solar cells, i.e., PEC-PVs, such as dye-sensitized solar cells (DSSCs) or perovskite PVs, has also been enthusiastically investigated and has produced comparable efficiency to that of the conventional solar cells reported on the laboratory scale^14^,^15^. PV panel modules generate a large amount of power and can be connected to a completely isolated electrolysis device after an adequate voltage conversion (“wired” PV + electrolyzer configuration). However, the substantial cost of PV module assembly and PV panel construction, as well as the complicated wiring system, imposes a critical barrier for scaling up a PV + electrolyzer system^16^,^17^,^18^.

It is therefore desirable to construct an effective, scalable, and simple water splitting system to directly achieve water splitting in one device. Many researchers have investigated powdered photocatalytic systems, which are considered a potentially scalable and commercially viable technology^19^,^20^. However, the currently achieved efficiencies with this technology remain low. Tremendous efforts have also been devoted to establish photoelectrochemical (PEC) processes, where solid-liquid interfaces are effectively utilized^4^,^8^,^21^. An efficient un-biased device has been reported using solar cells conjugated with electrolyzer (“unwired” PV + electrolyzer)^22^,^23^,^24^. Typically, in the “artificial leaf” configuration^25^,^26^, a cathode and an anode are separated by the semiconductor wafer itself, which causes a significantly high concentration overvoltage across the electrolysis cell. In some cases, an ionic membrane has been considered to be used to separate the (photo)anode and the (photo)cathode^27^, but it is expensive and often requires the use of an extreme pH. A membrane-less configuration^28^ reduces such overvoltage, as well as the overall system cost, although some associated problems have to be addressed, such as the co-generation of hydrogen and oxygen in the same electrolyte compartment resulting in the cross-over of product gases. The development of a simple and practical membrane-less water splitting system requires further detailed investigations^28^.

An efficient STH system requires low-overpotential electrocatalysis, regardless of the configuration. Thus, a selection of the electrocatalysts is crucial to optimize and maximize the overall efficiency^29^. Noble-metal electrocatalysts, such as IrO_x_^30^,^31^ or RuO_x_^32^,^33^ for the oxygen evolution reaction (OER) and Pt^34^,^35^ for the hydrogen evolution reaction (HER), are known to exhibit significantly high activities. Their limited resources and high costs, however, hamper their practical and large-scale usage. In the past decades, a variety of electrocatalysts from ubiquitous elements have been reported, such as CoO_x_ (in near-neutral condition) and NiFeO_x_ (in alkaline condition) for OER^36^,^37^,^38^,^39^ and metal phosphide (in acid) and NiMo, NiW, or NiFe (in alkaline condition) for HER^40^,^41^,^42^,^43^. Most of the works have been carried out under kinetically favored and extremely acidic or alkaline pH conditions, which raises issues such as the mechanical and chemical strengths of the material. Thus, earth-abundant and cost-effective electrocatalysts that are active under near-neutral pH aqueous conditions are highly desired for practical and industrial applications^37^.

The above discussion leads to a proposal of a simple semiconductor device generating a sufficient voltage for water electrolysis that is attachable with ubiquitous electrocatalysts. As one of the candidates for efficient and space-saving solar cells, spherical silicon has been reported, consisting of a solid-state p-n junction between the inside and the outside of the sphere^44^,^45^,^46^. They are usually synthesized by a dropping method from inexpensive and low quality materials, which make it possible to reduce the cost in the cutting or polishing process of silicon ingots^44^,^45^ and raw silicon materials itself. Because the spherical silicon generates photocurrents up to approximately 1 mA and approximately 0.6 V of *V*_OC_ with a relatively small volume^45^,^46^, the required voltage for the electrode reaction is easily adjusted by connecting them in series, e.g., a connection of 3–4 series for water splitting, without requirement of too large occupation area. Additionally, the spherical PVs can collect a much larger number of photons than flat plate solar cells because it can receive light from all directions. This feature realizes one advantage that the spherical PVs can maximize its performance for longer time in the daytime than flat plate PVs without adjusting its angle to receive the sunlight perpendicularly. Devices utilizing spherical silicon solar cells with semi-concentration reflectors have been reported^47^,^48^, indicating a possibility to construct space-saving and highly efficient PV devices when attached to suitable electrocatalysts.

In this report, a stand-alone and integrated PV device consisting of nickel-based electrocatalysts and a “SPHELAR” solar module^46^ was investigated as an efficient, compact, and scalable “PV + electrolyzer” water splitting system. The SPHELAR modules were composed of either three or four series-connected silicon balls protected with a polymer (3PVs or 4PVs; 0.20 cm^2^ and 0.26 cm^2^ for the projected surface area, respectively) as shown in the schematic diagrams of Fig. 1, which provided a sufficient working voltage for water electrolysis (1.5–2.0 V). The PV device was investigated in various configurations, i.e., SPHELAR fixed within or above the electrolyte solution and with or without a proper reflector. Based on these comprehensive configuration evaluations, a theoretical optimization of the electrocatalyst surface area and a proposal of a possible device design were addressed. We also discussed the physical properties associated with the separation of the evolved hydrogen and oxygen gases in the membrane-less system with an appropriate design of the whole apparatus. Our detailed electrochemical study showed that the non-noble metal-based electrodes (NiMo for the cathode and NiFe for the anode) provided a sufficiently high current at an alkaline pH for the 3PV configuration. Additionally, with NiCoO_x_ as the anode, a sufficient current for the 4PV configuration was achieved at a near-neutral pH. The presented device made from cost-effective materials exhibited STH efficiencies higher than 7%. This study was able to clearly demonstrate a novel approach to generate hydrogen by harvesting sunlight effectively with the completely stand-alone device.

## Results

### PV optimization: Managing light absorption by controlling scattering, reflection and absorption

Because the spherical-shaped SPHELAR PV module receives irradiated light from all directions, the PV conjugated with some reflector that collects scattered and/or reflected photons are expected to improve the photoconversion efficiency^47^,^48^. Compared with conventional thin film approaches, such module is much simpler. Additionally, this spherical shape of the SPHELAR PV module is definitely a great advantage over the conventional flat plate structure, because it can maximize the light irradiation for longer time in the daytime in spite of the daily sunlight angle change. In this study, a micro-foamed reflective sheet (MCPET; Furukawa Electric Co. Ltd.) was utilized as a reflector for the SPHELAR module. Figure 2 compiles the measured diffuse reflectance of MCPET and a mirror. The reflectance of MCPET was higher than the mirror above approximately 430 nm, implying that MCPET was capable of reflecting and scattering a larger number of photons in most of the visible and near infrared range than a common mirror. This observation suggests that use of MCPET as a reflector would greatly enhance the photocurrent.

When the SPHELAR module is placed in water, the surrounding water markedly changes the refractive index optimized for the configuration in air, which in turn affects its light absorption due to reflection, scattering, and absorption by the water. Nevertheless, light-absorbing semiconductors may have to reside in water for a stand-alone water splitting system. The absorption coefficient of pure water was investigated both theoretically^49^ and experimentally^50^,^51^, which revealed that photons were absorbed not only in the infrared region but also in the visible light region to a different degree, as shown in Supplementary Fig. S1. The solar spectra at different depths of pure water were calculated with the absorption coefficient of water and are illustrated in Fig. 3, where the integrated values of the photon flux at each water depth are summarized as a percentage relative to AM1.5G. Above approximately 500 nm, the presence of water significantly reduced the irradiance inside the water. In the infrared region, only a water depth of 5 cm was required to absorb most of the photons (0.07 decreased to approximately 0.01 mW cm^−2^ nm^−1^), which would decrease the available amount of photon for the PVs in water. Particularly in the case of using silicon as a light absorber, light absorption by water seems to be a crucial issue because silicon utilizes infrared light. Although the absorption coefficient of a liquid depends on the identity of the electrolytes^52^,^53^, light absorption by water is very critical in the scale-up development of stand-alone water splitting devices when light absorbers are placed in water, such as integrated PEC systems. In such cases, the depth of water should be adequately designed to be as low as possible to lower the light absorption by electrolytes, or wherever possible, it is desirable to locate the PV above water while maintaining the connections with the electrodes that are immersed in water. Notably, these effects of light condensation by a reflector and photon loss due to water can be regarded as one of the most crucial factors to establish an efficient STH production system. In a “PV + electrolyzer” system, the photon collection efficiency can be easily improved when the light absorbers and catalysts are separated. This aspect is a great advantage over other solar hydrogen production systems. In the following section, various configurations (PVs fixed in/above water) are investigated.

**Figure 1:**
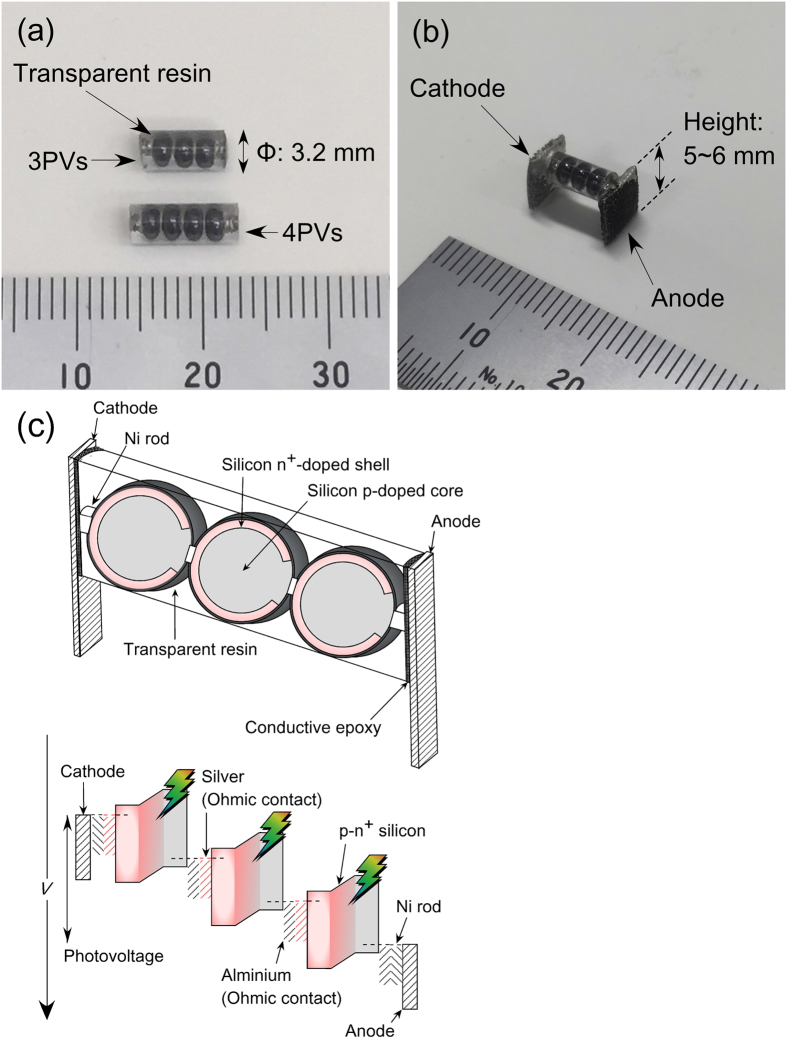
Photographs of SPHELAR devices. (**a**) The series connected spherical silicon solar cells molded in transparent resin with 3.2 mm of diameter (SPHELAR, Kyosemi co. ltd.), the three series of silicon (3PVs, upper) and four series of silicon (4PVs, bottom), whose lengths of the body were approximately 0.7 and 0.9 cm, respectively, (**b**) the stand-alone module of 3PVs with electrocatalysts of minimized surface area. Scale: mm, and (**c**) schematic diagrams for SPHELAR module.

**Figure 2:**
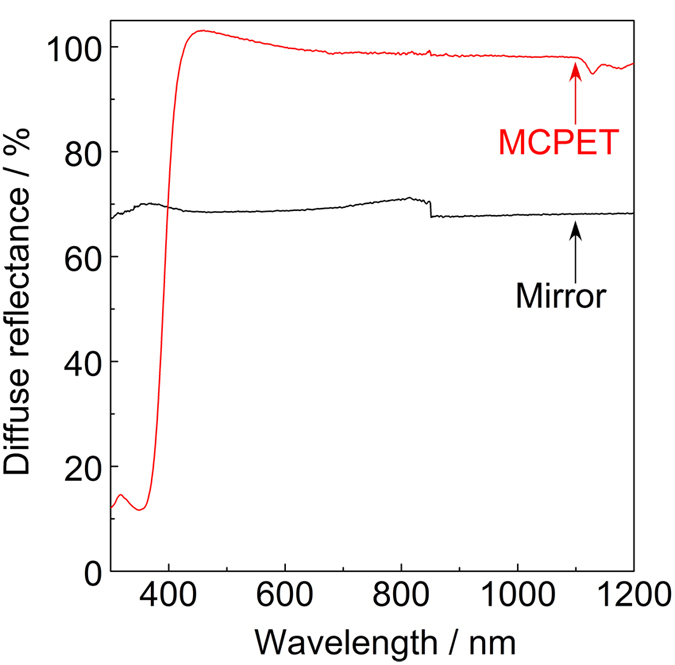
Diffuse reflectance spectra for reflectors. Red line indicates microfoamed reflective sheet (MCPET, Frukawa electric co. ltd.) and black line means stainless mirror with wavelength of 300–1200 nm.

**Figure 3:**
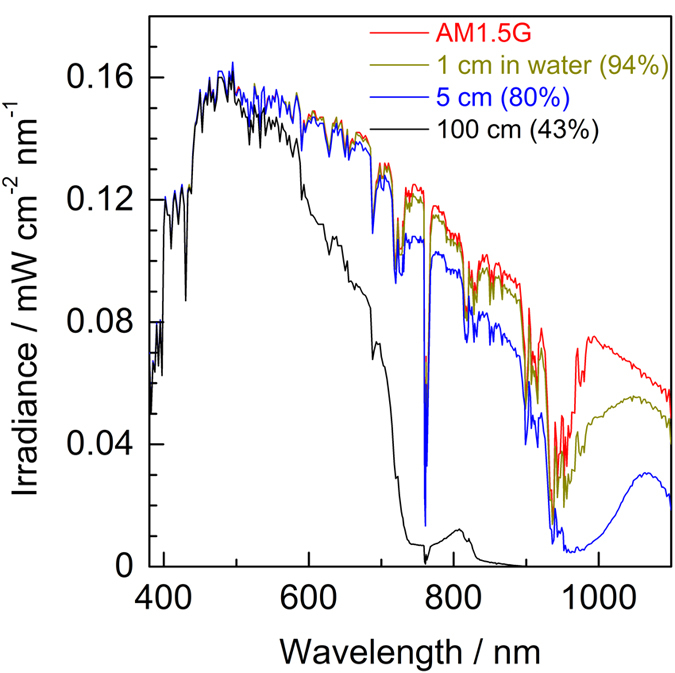
Solar irradiance at different depth of pure water (22 °C) calculated from the reported absorption coefficient (Supplementary Fig. S1). AM1.5G (reported by NREL), solar spectra at 1, 5, and 100 cm depth of water are illustrated in red, yellow, blue, and black line, respectively. The integrated portions of photon flux (380–1100 nm) are designated in brackets as the percentage to pristine AM1.5G.

**Figure 4:**
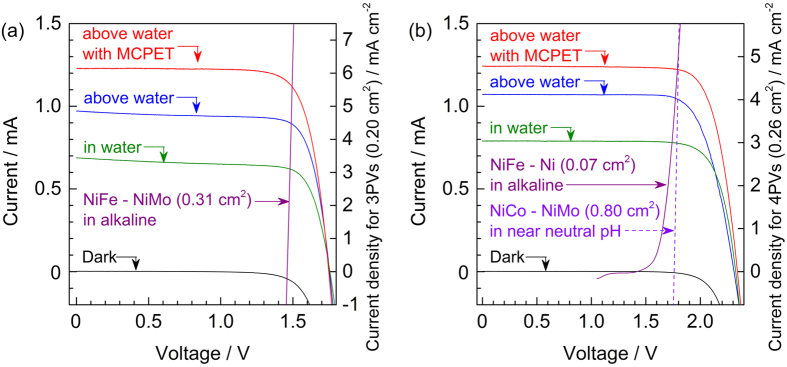
Current-voltage properties for SPHELARs and electrocatalysts. (**a**) 3PVs and (**b**) 4PVs were performed in various configurations, where SPHELAR was fixed at approximately 1 cm depth of water, above water, and above water with MCPET illustrated in green, blue, and red lines, respectively. As representative choices of electrocatalysts, *I–V* curves for 0.31 cm^2^ of NiFe – NiMo in alkaline solution for 3PVs and 0.80 cm^2^ of NiCo – NiMo in near neutral pH solution or 0.07 cm^2^ of NiFe – Ni in alkaline for 4PVs were also plotted. The second y-axis corresponds to the “current density” for each SPHELAR (3PVs and 4PVs), which means photocurrent generated by SPHELAR divided by projected surface area of SPHELAR. The voltage of SPHELAR was swept from open-circuit to short-circuit at 10 mV s^−1^, and the voltage of electrocatalysts was swept at 10 mV s^−1^ cathodically. As a light source, solar simulator adjusted to AM1.5G was used.

The current-voltage profiles of one SPHELAR (3PVs or 4PVs) module in various configurations, i.e., at a water depth of 1 cm, above water, and above water with MCPET, are shown in Fig. 4 and Supplementary Table S1. The 3PVs generated approximately 1.8 V of open-circuit voltage (*V*_OC_) and approximately 1.5 V of power maximum voltage (*V*_PM_), and the 4PVs exhibited 2.3 V for *V*_OC_ and 1.9 V for *V*_PM_. The voltage differences observed in 3PVs and 4PVs originated from the number of connected silicon cells. When placed above the electrolyte, one SPHELAR module generated approximately 1.0 mA of photocurrent at the plateau. With the MCPET reflector, the photocurrent was improved to >1.2 mA. In the SPHELAR module, there is a transparent resin surrounding spherical silicon balls as seen in Fig. 1, which is aimed at maximizing the absorption of light by the Si SPHELAR while suppressing the light reflection from the device. The difference in observed photocurrents with/without the MCPET implies that the total absorption of photons failed with a single path, and that the reflected light by the MCPET was further absorbed by the SPHELAR. In contrast, the SPHELAR module placed in the electrolyte exhibited a significantly smaller photocurrent of approximately 0.7–0.8 mA. Although a water depth of 1 cm absorbed only 6% of the photons in solar irradiance (Fig. 3), the photocurrent of SPHELAR in water decreased by approximately 20–30% compared with that placed above water. This might be due to not only the light absorption by water but also a decrease in the lens effect of the transparent polymer resin molding of SPHELAR. The light refracting angle from the water to the resin is expected to be smaller than that from the air to the resin because the refractive indices of air, water, and the resin are approximately 1.0, 1.3, and 1.5, respectively^54^,^55^,^56^. These observations quantitatively revealed the importance of the PV module location. The current module system was capable of generating a maximum photocurrent of approximately 1.2 mA even at a plateau region (up to 1.5 V and 1.9 V for 3PVs and 4PVs, respectively) corresponding to a STH efficiency higher than 7%, assuming that all the photocurrents were converted into hydrogen and oxygen.

### Electrocatalyst optimization: Tuning the catalyst identity and size for maximal performance

**Figure 5:**
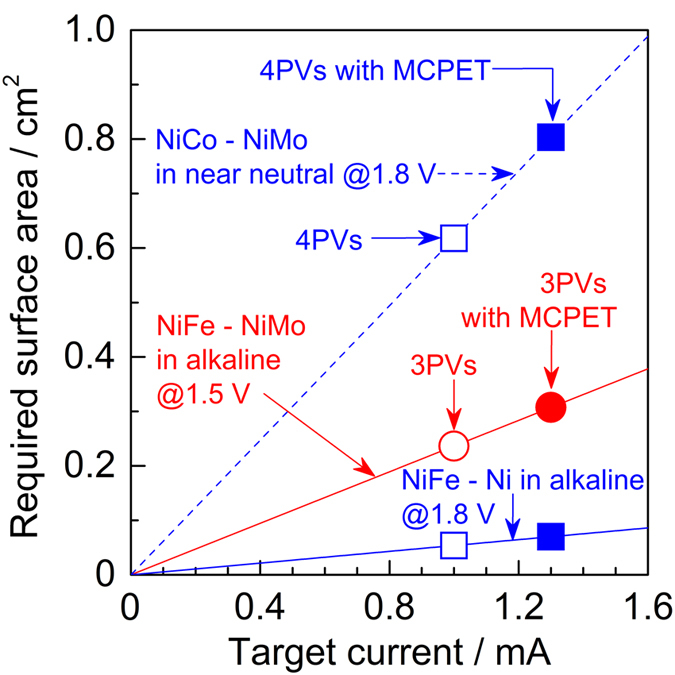
Theoretical required surface area for electrodes. Combinations of NiFe – NiMo in alkaline solution for 3PVs, red line; NiFe – Ni in alkaline for 4PVs, blue line; and NiCo – NiMo in near neutral pH solution for 4PVs, blue dashed line were illustrated as a function of target current at 1.5 V for 3PVs and 1.8 V for 4PVs calculated from current-voltage properties for SPHELARs (Fig. 4) and electrocatalysts (Supplementary Fig. S2). The required surface area for 3PVs, 3PVs with MCPET, 4PVs, and 4PVs with MCPET are illustrated by ○, ●, □, and ■, respectively.

The identity and amount of electrocatalysts should be determined to achieve the highest PV photocurrent. Although typical PV systems are designed to operate at the maximum power, the overall STH efficiency of a integrated “PV + electrolyzer” system is maximized by the utilization of the maximum photocurrent rather than achieving the maximum PV electric power^16^,^17^. Water electrolysis was examined over five combinations of electrocatalysts in 0.5 M KOH: (Anode, Cathode) = (NiFe, NiMo), (NiFe, Pt/Ni), (NiFe, Ni), (Ni, Pt/Ni) and (Ni, Ni). Additionally, four combinations were evaluated for water electrolysis in 1.5 M K-phosphate (pH 5.8; a mixture of 1.5 M KH_2_PO_4_ and 1.5 M K_2_HPO_4_ with 80:20 ratio)^57^: (NiCo, NiMo), (Ni, NiMo), (NiCo, Ni) and (Ni, Ni). Supplementary Fig. S2 summarizes the *j*-*E* curves for all catalyst combinations. In all cases, the observed current densities increased with the applied voltage. In the alkaline solution, (NiFe, NiMo) and (NiFe, Pt/Ni) showed the smallest onset voltage of approximately 1.5 V, followed by (Ni, Pt/Ni), (NiFe, Ni) and (Ni, Ni). Our separate studies of electrolysis at a near-neutral pH revealed that a maximal performance was attained in a mixture of 1.5 M KH_2_PO_4_ and 1.5 M K_2_HPO_4_ with 80:20 ratio^57^. Nevertheless, the required voltage to achieve water electrolysis, even in the optimized phosphate solution, was found to be larger than those in the alkaline solution. In the K-phosphate electrolyte solutions, (NiCo, NiMo) and (Ni, Ni) showed the highest and smallest current densities at a given overvoltage, respectively. The polarization curves for (NiCo, Ni) and (Ni, NiMo) appeared between (NiCo, NiMo) and (Ni, Ni). As discussed in the previous section, the operating voltages for SPHELARs were below 1.5 V (3PVs) and 1.9 V (4PVs) with the maximum photocurrent of approximately 1.2 mA at the plateau. The electrochemical study revealed that 1.2 mA at such overvoltage was achievable with (NiFe, Pt/Ni) and (NiFe, NiMo) for 3PVs and (NiFe, Ni) for 4PVs in alkaline solution. Additionally, (NiCo, NiMo) and (NiCo, Ni) were found to be capable of catalyzing water electrolysis at a maximum PV photocurrent below 1.9 V (4PVs). The minimum electrode geometric surface area *SA*_*min*_, which was required to achieve the maximum photocurrent at the operating voltage, was calculated to optimize the system for (NiFe, NiMo) with 3PVs in an alkaline solution and (NiCo, NiMo) with 4PVs in a near-neutral pH solution. The following relationship was used:


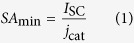


where *I*_SC_ is the short-circuit current (achievable maximum photocurrent by the PVs) and *j*_cat_ represents the electrocatalytic current density at the operating voltage (at which the *I*_*SC*_ is obtained). *I*_*SC*_ was adopted from the *I*-*V* curves for the SPHELARs (Fig. 4), and *j*_*cat*_ was obtained from the *j*-*E* curves for the electrocatalysts (Supplementary Fig. S2). In Fig. 5, the calculated minimum electrode surface area is compiled against the electric current, where the inverse of the slope (mA cm^−2^) corresponds to the current density over the electrode at the operating voltage. The y-value at the target current, *I*_SC_, corresponds to the *SA*_min_ needed to obtain *I*_*SC*_. It should be noted that the surface areas of both the anode and the cathode were the same. The calculated *SA*_min_ for each electrode configuration is summarized in Supplementary Table S2. The power-voltage relationship for the PV and water electrolysis with the optimum electrode surface area is compared in Supplementary Fig. S4.

Generally, the electrolysis process contains a solution resistance that induces an additional voltage requirement especially at high current operations^8^,^58^. The resistance *R* is theoretically described by the following equation:


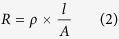


where *ρ* is the resistivity; and *l* and *A* are the specific length and area, respectively. For the solution resistance in an electrochemical cell, the ratio of *l*/*A* is reduced to the cell constant (*K*_*cell*_). This theoretical aspect suggests that solution resistance increases with increasing distance between the electrodes and with decreasing electrode surface area. Here, the solution resistances (or cell constants) and the resultant *iR* drops were quantitatively investigated in 0.5 M KOH with various electrode surface areas and distances by electrochemical impedance spectroscopy. The measured solution resistances and cell constants were discussed in the Supporting Information (Supplementary Fig. S6). Based on our comprehensive benchmark study on the cell constant in various configurations (distance, height and width), cell constants and solution resistances in our present SPHELAR devices were calculated to be 10.3 mV (0.45 cm^2 ^in 3PVs) and 13.8 mV (0.19 cm^2^ in 4PVs). More details are found in the Supporting Information (Supplementary Fig. S7). Notably, as seen in Equation 2, the solution resistance varies by the type of electrolyte. The detailed analysis on solution resistivity was reported previously^57^,^59^,^60^. According to the report, the K-phosphate used exhibited the minimal solution resistivity. Overall, in the SPHELAR system configuration, a reduction of the cell constant and resultant optimal solution resistance can be readily achieved due to the short distance between electrodes that are directly attached to both ends of the small SPHELAR module (~9 mm).

### Overall water splitting by SPHELAR in various configurations

**Figure 6:**
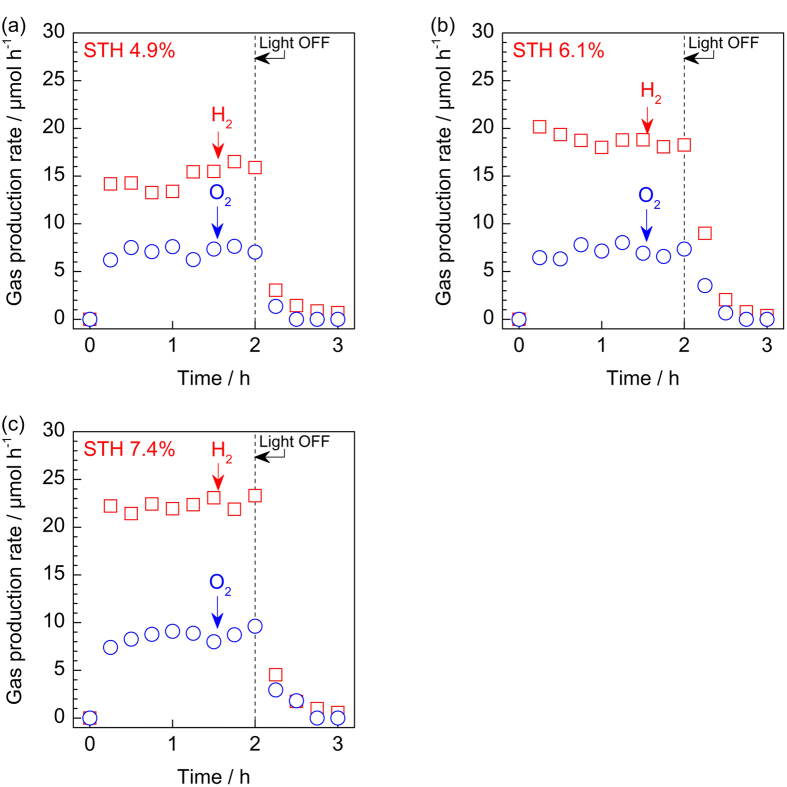
Time courses of overall water splitting into H_2_ (☐) and O_2_ (○) by 3PVs. Overall water splitting were demonstrated by (**a**) 3PVs fixed in 0.5 M KOH aqueous solution as an electrolyte, (**b**) 3PVs fixed above the electrolyte, and (**c**) 3PVs with MCPET fixed above the electrolyte under irradiation of solar simulator (AM1.5G). As the electrocatalysts, combination of NiFeO_x_ (anode) and Pt/Ni (cathode) was used with geometric surface area of approximately 0.34 cm^2 ^and 0.45 cm^2^ for 3PVs (**a**,**b**) and 3PVs with MCPET in air (**c**), respectively. The reactions were measured in a Pyrex top-irradiation flow cell at room temperature.

**Figure 7:**
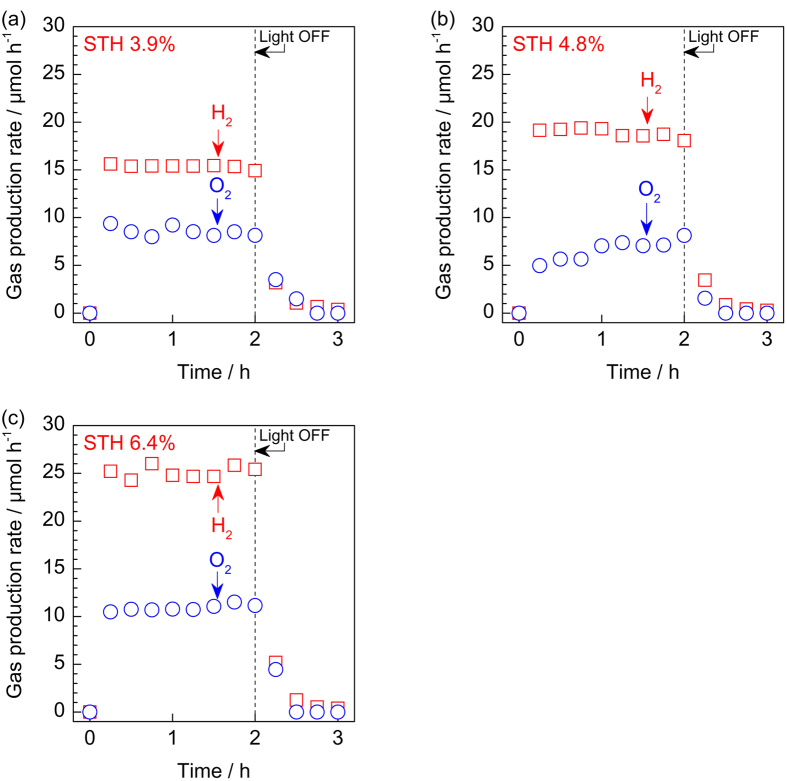
Time courses of overall water splitting into H_2_ (☐) and O_2_ (○) by 4PVs. Overall water splitting were demonstrated by (**a**) 4PVs fixed in 0.5 M KOH aqueous solution as an electrolyte, (**b**) 4PVs fixed above the electrolyte, and (**c**) 4PVs with MCPET fixed above the electrolyte under irradiation of solar simulator (AM1.5G). As the electrocatalysts, combination of NiFeO_x_ (anode) and Ni (cathode) was used with geometric surface area of approximately 0.14 cm^2^ and 0.19 cm^2^ for 4PVs (**a**,**b**) and 4PVs with MCPET in air (**c**), respectively. The reactions were measured in a Pyrex top-irradiation flow cell at room temperature.

**Figure 8:**
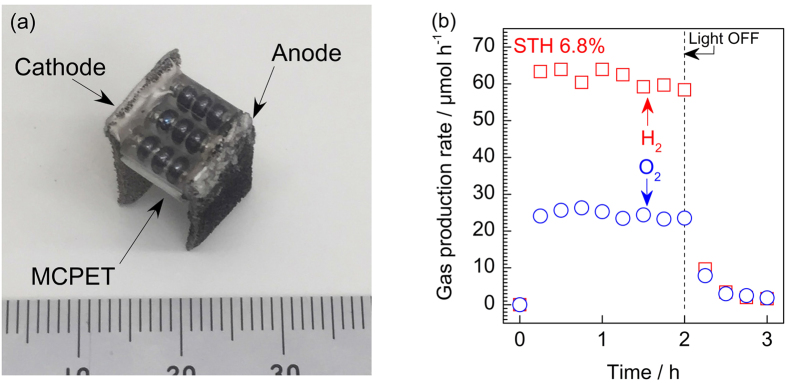
Potential method for the scale-up of the SPHELAR device. (**a**) A photograph of the demonstration for a scaled up SPHELAR device consisting of three parallel of 3PVs with MCPET, whose projected area were considered to be 0.6 cm^2^ for STH calculation. (**b**) Time course of overall water splitting into H_2_ (□) and O_2_ (○) by three parallel of 3PVs (**a**) fixed above 0.5 M KOH aqueous solution as an electrolyte under irradiation of solar simulator (AM1.5G). As the electrocatalysts, combination of NiFeO_x_ (anode) and Pt/Ni (cathode) was used with geometric surface area of approximately 1.35 cm^2^. The reactions were measured in a Pyrex top-irradiation flow cell at room temperature.

In this section, overall water splitting with our SPHELAR devices, composed of (NiFe, Pt/Ni) for 3PVs and (NiFe, Ni) for 4PVs in 0.5 M KOH, are demonstrated as model cases. The electrode surface areas were 0.45 cm^2^ for 3PVs with MCPET and 0.19 cm^2^ for 4PVS with MCPET (see Supplementary Fig. S5 for calculation details). Various SPHELAR configurations were evaluated, i.e., the body of SPHELAR was fixed in the electrolyte, above the electrolyte, and above the electrolyte with MCPET. Figs 6 and 7 show the measured gas production rate by devices with 3PVs and 4PVs, respectively, where the corresponding STH efficiencies calculated with the PV module projected surface area are also noted. The measured hydrogen evolution rates and STH efficiencies are summarized in Supplementary Table S3. In all cases, steady evolutions of hydrogen and oxygen were observed during light irradiation. The light was turned off after 2.0 h, and no further formations of hydrogen and oxygen were observed, thus confirming that gas productions were induced by harvesting the light. Additionally, it was confirmed that there was no consumption of evolved hydrogen and oxygen in the circulation system after the light was turned off (shown in Supplementary Fig. S8), indicating a negligible reverse reaction of products forming water in the dark. In both measurements in the flow reactor (Figs 6 and 7) and in the batch reactor (Supplementary Fig. S8), the ratio of evolved hydrogen over oxygen was approximately 2, which agreed with the stoichiometry of the water splitting reaction. The devices with MCPET showed the highest STH efficiencies of 7.4% (3PVs) and 6.4% (4PVs). Importantly, the STH efficiency was calculated with the projected surface area of the SPHELAR device and the same area of the incident photons. Therefore the STH efficiency improved with the MCPET reflector, which successfully utilized the photons unused unless it is present, as demonstrated in the previous section. Without MCPET as the reflector, smaller efficiencies were obtained: 6.1% (3PVs) and 4.8% (4PVs) with SPHELAR above the electrolyte and 4.9% (3PVs) and 3.9% (4PVs) with SPHELAR in the electrolyte. The differences in the STH efficiencies due to device location can be attributed to light absorption by the electrolyte and the altered lens effect, as shown in Figs 3 and 4 54,55,56. The hydrogen and oxygen production rates in Figs 6 and 7 agreed with the expected photoelectric and electric currents (see Fig. 4), revealing that an approximately 100% of Faradic efficiency was attained. Indeed, a spontaneous gas evolution from both the anode and the cathode could be seen in the supplementary Movie S1 using a representative configuration of 3PVs located above the electrolyte with MCPET. As clearly seen in the supplementary movie, this SPHELAR based device could split water only by solar energy and work stand-alone without any bias or extra circuit. As Fig. 1(a) demonstrates the actual photograph of SPHELARs, the total volumes for SPHELARs were approximately 5.6 × 10^−2^ and 7.2 × 10^−2 ^cm^3^ for 3PVs and 4PVs, respectively. Since the present SPHELAR devices could split water in the completely stand-alone configuration, it is remarkable that such a small volume (less than 0.1 cm^3^) of the present PV + electrolyzer system achieves the high STH efficiencies. Additionally, a potential method for the scale-up of the SPHELAR device, where three 3PVs are placed in parallel with MCPET and shared the electrocatalyst, was demonstrated. The picture of this configuration is shown in Fig. 8(a), which can also show how the reflector was integrated in the SPHELAR device. The projected surface area of the whole device was approximately 0.6 cm^2^, which was three times larger than that of one 3PV module (0.20 cm^2^). The amount of evolved gas upon irradiating the photon is compiled in Fig. 8(b). Indeed, an approximately three times larger gas production rate was observed in the configuration compared with one 3PV module. The observed hydrogen production rate corresponded to approximately 6.8% of STH efficiency. This STH efficiency was slightly smaller than that of one 3PV (ca. 7.4%), which was likely due to a scramble of the scattered lights by the MCPET between the SPHELAR modules. Nonetheless, the demonstrated device with three parallel SPHELARs showed a quite promising scale-up capability with a STH efficiency higher than 5%.

In this study, a simple “PV + electrolyzer” system has been demonstrated. It is true that the membrane-less system, where both hydrogen and oxygen are produced in the same electrolyte compartment, can potentially induce the cross-over of product gases during long time operation or industrial scale-up. Nevertheless, this issue can be addressed by a proper design of the overall apparatus, e.g., the construction of a gas channel over the electrodes, which provides merely a physical driving force without any other gas separation and purification processes. Overall, the current study proposed an entirely stand-alone and scalable integrated “PV + electrolyzer” system composed of earth-abundant materials with a high STH efficiency (≥5%). The efficiency of the module was successfully optimized in this report by 1) the generation of a large photocurrent in a small occupied surface area, 2) collection of the irradiated light by a proper reflector, and 3) reduction of catalyst amounts by theoretical optimization. The STH efficiency can be further enhanced by simply improving PV efficiency in the SPHELAR module.

## Discussion

A novel “PV + electrolyzer” system consisting of spherical silicon solar cells connected in series, named “SPHELAR,” and conjugated with electrocatalysts was investigated for solar hydrogen production. SPHELAR composed of 3PVs and 4PVs generated open-circuit voltages (*V*_OC_) of approximately 1.8 V and 2.3 V, respectively. A maximum photocurrent of 0.7–0.8 mA was observed with an occupation area of 0.20 cm^2^ (3PVs) or 0.26 cm^2^ (4PVs) when the SPHELAR was dipped in water. An approximately 30% increase in the photocurrent was attained by placing the device above water, which was likely due to the avoidance of photon absorption by the water and an increase in the lens effect by the molding resin. The photocurrent was further enhanced by 20% by placing a micro-foamed reflective sheet underneath the device owing to a successful reflection of light that is reflected and/or scattered by the silicon balls. The largest operating voltages which can generate the maximum photocurrent (*V*_op_) by the SPHELAR were found to be 1.5 V and 1.8 V for 3PVs and 4PVs, respectively. Various electrodes were separately investigated for water electrolysis, i.e., NiFe, Ni and NiCo for the anode and Pt/Ni, NiMo and Ni for the cathode. (Anode, cathode = NiFe, Pt/Ni) and (NiFe, NiMo) showed onset voltages smaller than *V*_op_ for 3PVs, and even the (NiFe, Ni) combination exhibited a sufficiently lower overvoltage than *V*_*op*_ for 4PVs in 0.5 M KOH. Furthermore, the (NiMo, NiCo) combination was found to be capable of catalyzing water electrolysis at voltages smaller than *V*_*op*_ for 4PV at a near-neutral pH. By effectively comparing the *I*–*V* curves for PV and the electrocatalysts, the maximum photocurrent of PVs was achieved with an electrode surface area less than 0.5 cm^2^. Such a relatively small electrode occupation area prevented the shielding of irradiated light by the electrodes. Finally, using SPHELAR conjugated with the electrodes, completely stand-alone solar-driven overall water splitting was demonstrated. In all cases, a stoichiometric gas evolution and approximately 100% of Faradic efficiency were attained. The most efficient configuration exhibited 7.4% of STH efficiency when 3PVs with MCPET was located above the electrolyte. A potential scale-up approach was also provided by simply sharing the electrodes with three 3PVs placed in parallel with MCPET, which successfully achieved a STH efficiency of approximately 6.8%. Notably, the relatively small size of SPHELAR made distances between the electrodes shorter, which provided a quite small *iR* drop in the system (accounted for less than 1% of the overall voltage). Overall, in the present study, a novel “PV + electrolyzer” approach, which was completely stand-alone, efficient, compact, and easily scalable, was successfully demonstrated with the “SPHELAR” module and the proper combination of electrocatalysts. This work provided a new reasonable approach for solar fuel production.

## Methods

### Material preparation

The series-connected spherical silicon solar cells molded in a transparent resin, named “SPHELAR,” were manufactured by Kyosemi Co. Ltd. Fig. 1 shows a picture of SPHELAR. The series spherical silicon was connected to a Ni rod electrode with an ohmic contact, and its schematic energy diagrams are illustrated in Fig. 1(c). Projected surface areas of SPHELAR consisting of 3 series of silicon (3PVs) and 4 series of silicon (4PVs) were approximately 0.20 cm^2 ^and 0.26 cm^2^, respectively. These areas were used for the STH efficiency calculations. Microfoamed reflective sheets (MCPET; Furukawa Electric Co. Ltd.), as a reflector for SPHELAR, were prepared in the same size as the body of SPHELAR.

For the electrochemical investigation, a Ni foam was used as a substrate and also as an electrocatalyst itself. NiFe, as an anode, was prepared by hydrothermal synthesis^5^. A Ni foam was immersed in an aqueous solution containing 1 mM of Ni(NO_3_)_2_ 6H_2_O (99.999%, Sigma-Aldrich), 1 mM of Fe(NO_3_)_3_ 9H_2_O (99.99%, Aldrich), and 5 mM of urea (>99.5%, Sigma-Aldrich). The sample was heat treated at 393 K for 12 h in an autoclave. NiCo electrocatalysts were prepared by electrochemical deposition. A Ni foam with a geometric size of 1 × 1 cm was immersed in a 0.1 M Co nitrate aqueous solution (Co(NO_3_) 6H_2_O, 99.999%, Aldrich), and an electric current of −10 mA was applied for 1 h. For the Pt/Ni electrode as a cathode, Pt was deposited on Ni foam with a thickness of approximately 6 nm by RF-magnetron sputtering. NiMo electrocatalysts were electrochemically deposited onto the Ni foam^57^. A Ni foam with a geometric size of 1 × 1 cm was immersed in the bath containing 0.02 M sodium molybdate (Na_2_MoO_4_ 2H_2_O, 99.99%, Sigma-Aldrich), 0.04 M nickel chloride (NiCl_2_ 6H_2_O, Sigma-Aldrich) and 0.89 M sodium bicarbonate (NaHCO_3_, 99.5–100.5%, Sigma-Aldrich). An electric current of −77.5 mA was applied to the Ni foam for 30 min. The obtained NiMo electrode was kept in a 0.5 M KOH solution for 15 h. The SEM images of the electrodes (Ni foam, NiMo, and NiFeO_x_) are shown in the Supplementary Fig. S3^61^. For the following gas analyses, the prepared Pt/Ni or Ni as the cathode and NiFe or Ni as the anode were connected to both edges of the SPHELAR by conductive epoxy resin (ITW Chemtronics Co. Ltd., CircuitWorks Conductive Epoxy) to evaluate the integrated system, as shown in Fig. 1(b).

### Electrochemical measurements of the electrocatalysts and PVs

Water electrolysis was investigated in the two-electrode system with Ar bubbling and without stirring. Surface areas of both anode (NiFe, NiCo, or bare Ni foam) and cathode (Pt/Ni, NiMo, or bare Ni foam) were 1 × 1 cm in geometric size. The electrochemical performances over NiFe, Pt/Ni, NiMo, and Ni were evaluated in a 0.5 M KOH aqueous solution (pH ≈ 13.8), whereas those at near-neutral pH were in 1.5 M K-phosphate (KH_2_PO_4_/K_2_HPO_4 _= 80/20, pH 5.8)^57^ over NiCo, NiMo, and Ni electrodes. The applied voltage was scanned at −10 mV s^−1^. The solution resistances were comprehensively measured in various configurations, i.e., various widths (1–4 cm), heights (1–3 cm) and distances (1.5–8 cm) of Ni electrodes, by electrochemical impedance spectroscopy (100 kHz and 10 mV amplitude). All *i*–*E* relationships in this study are reported without *iR*-correction.

The current-voltage properties for SPHELAR (3PVs and 4PVs) were also investigated by the two-electrode system with a scan rate of 10 mV s^−1^ from open-circuit to short-circuit. As a light source, a simulated sunlight (AM1.5G) with irradiation area of approximately 60 × 60 mm was applied. During the measurements, the bodies of SPHELAR were fixed in various positions: A) At a depth of 1 cm in water, B) above water (in air), and C) above water with MCPET.

### Gas analyses for overall water splitting

The demonstration of overall water splitting by SPHELAR (3PVs and 4PVs) conjugated with electrocatalysts was performed in a Pyrex top-irradiation flow cell with 50 SCCM of Ar flow at room temperature in a 0.5 M KOH aqueous electrolyte solution (pH ≈ 13.8). The bodies of SPHELAR were fixed A) in the electrolyte, B) above the electrolyte, and C) above the electrolyte with MCPET, in a similar manner to the previous section. NiFe and Pt/Ni were used as the anode and the cathode (NiFe, Pt/Ni), respectively, in the 3PV system, and (NiFe, Ni) was used in the 4PV system. The geometric surface areas of these electrocatalysts were adjusted to achieve the maximum current of the SPHELAR at operating voltages (same geometric size for both the anode and the cathode; details are discussed in the Supporting Information: Supplementary Fig. S5). A simulated sunlight (AM1.5G) with irradiation area of approximately 60 × 60 mm was used as a light source (see Supplementary Fig. S9 for the irradiance). Prior to the measurement, the reactor was purged with vigorous Ar flow for dozens of minutes until the remaining air was completely removed. The evolved gases were analyzed by micro-gas chromatography (Agilent Technologies Co. Ltd., 490 Micro GC) equipped with a TCD detector and a MS-5A column using Ar as a carrier gas. The demonstration for overall water splitting was also performed in a Pyrex top-irradiation cell connected to a recirculating batch reactor at room temperature, where evolved gases were analyzed by gas chromatography (Shimadzu Co. Ltd., GC-8A) equipped with a TCD detector and a MS-5A column using Ar as a carrier gas to confirm that consistent data can be obtained in both batch and flow systems, as shown in Supplementary Fig. S8.

### Characterization of micro-foamed reflective sheets

The reflectance and scattering properties of micro-foamed reflective sheets (MCPET) were studied by UV-vis diffuse reflectance spectroscopy (JASCO Co. Ltd., V-670 spectrophotometer). As a comparison, a diffuse reflectance spectrum of a stainless mirror was also measured under the same conditions.

## Additional Information

**How to cite this article**: Kageshima, Y. *et al*. A miniature solar device for overall water splitting consisting of series-connected spherical silicon solar cells. *Sci. Rep*. **6**, 24633; doi: 10.1038/srep24633 (2016).

## Supplementary Material

Supplementary Information

Supplementary Video S1
