# Supplementary material for: A miniature solar device for overall water splitting consisting of series-connected spherical silicon solar cells
Source: Sci Rep. 2016 Apr 18;6:24633. doi: 10.1038/srep24633 (PMC4834539; doi:10.1038/srep24633)
Supplement: Supplementary Information [file srep24633-s1.pdf]

# Supplementary Information

# A miniature solar device for overall water splitting consisting of series-connected spherical silicon solar cells

Yosuke Kageshima[1*], Tatsuya Shinagawa[2*], Takaaki Kuwata[3], Josuke Nakata[3], Tsutomu Minegishi[1], Kazuhiro Takanabe[2] & Kazunari Domen[1]

[1]Department of Chemical System Engineering, The University of Tokyo, 7-3-1 Hongo, Bunkyo-ku, Tokyo, 113-8656, Japan
[2]Division of Physical Science and Engineering, KAUST Catalysis Center (KCC), King Abdullah University of Science and Technology (KAUST), 4700 KAUST, Thuwal, 23955-6900, Saudi Arabia
[3]Kyosemi Corporation, 949-2 Ebisu-cho, Fushimi-ku, Kyoto, 612-8201, Japan

[*]These authors contributed equally to this work.
Correspondence and requests for materials should be addressed to K.T. (email: kazuhiro.takanabe@kaust.edu.sa) or to K.D. (email: domen@chemsys.t.u-tokyo.ac.jp).

**Theoretical and experimentally-investigated absorption coefficients of pure water**

Supplementary Fig. S1 shows the reported experimental values of the absorption coefficient of pure water, which correspond exactly to the theoretical calculation. The solar spectra at different depths of pure water were determined from these reported values (Fig. 3).

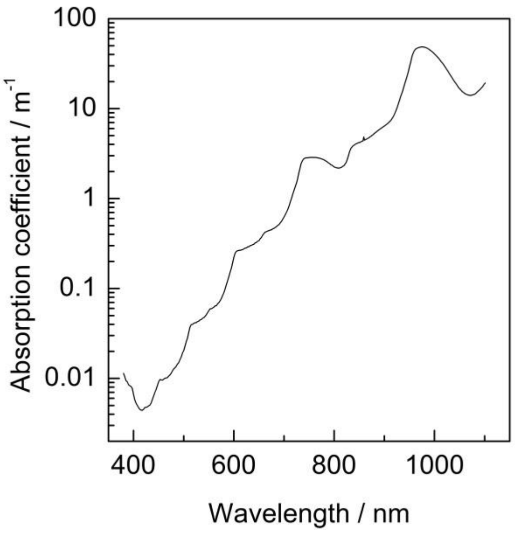


**Supplementary Figure S1.** The absorption coefficient of pure water (22 °C) as a function of wavelength previously reported experimentally (referred in the main manuscript).

**Current-voltage profiles for various electrocatalyst combinations**

The *J-V* properties in water splitting for the different electrocatalyst combinations discussed in the manuscript are illustrated in Supplementary Fig. S2. (Anode, cathode) = (NiFe, NiMo), (NiFe, Pt/Ni), (Ni, Pt/Ni), (NiFe, Ni), and (Ni, Ni) were evaluated in an alkaline solution (0.5 M KOH), while (NiCo, NiMo), (Ni, NiMo), (NiCo, Ni), and (Ni, Ni) were examined in a near-neutral solution (1.5 M K-phosphate: $KH_2PO_4/K_2HPO_4$=80/20, pH 5.8). The *J-V* curves were plotted without *iR* correction. Because the anodic scan itself includes both the oxidation and reduction of the catalyst materials to some extent, the cathodic scan, which reflects the catalytic currents that are consistent with chronoamperometric measurements, was mainly represented. In the alkaline solution, non-noble electrocatalysts, such as (NiFe, NiMo) for 3PVs or (NiFe, Ni) for 4PVs, were found to be sufficiently active and stable at the PV operating voltages of approximately 1.5 and 1.9 V for 3PVs and 4PVs, respectively. Also in the near-neutral solution, the non-noble metal catalysts, such as (NiCo, NiMo), exhibited smaller onset voltages than the 4PV photovoltage of 1.8-2.0 V. Thus, a combination of non-noble metal electrocatalysts can obviously be used in a near-neutral solution.

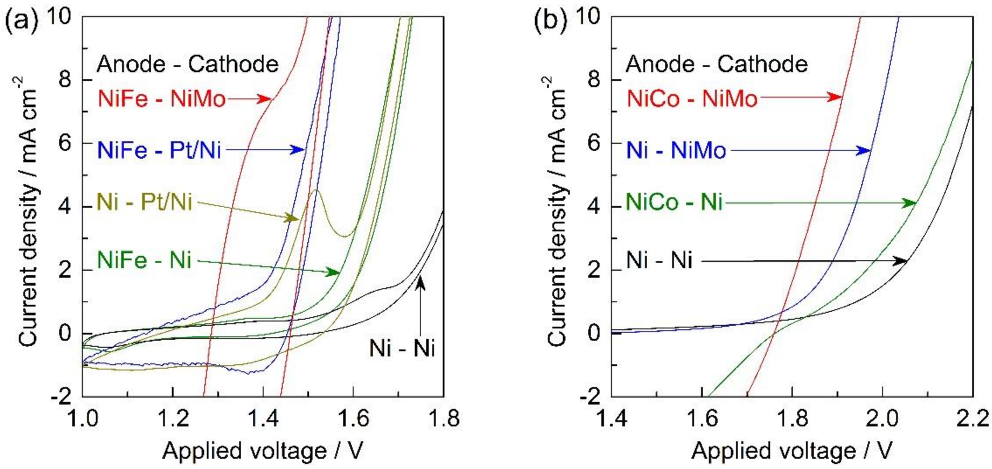


**Supplementary Figure S2.** Current-voltage properties for the combinations of electrocatalysts in (a) alkaline solution and (b) near neutral solution with a scan rate of -10 mV $s^{-1}$. 0.5 M KOH (pH 13.8) and 1.5 M K-phosphate ($KH_2PO_4/K_2HPO_4$=80/20, pH 5.8) were used as a alkaline solution and near neutral solution, respectively. All results were plotted without *iR* correction.

**SEM images for electrocatalysts**

The surface morphologies for Ni foam substrate, NiMo cathode, and NiFe anode were provided in the following SEM images. The SEM image for Ni foam substrate in Supplementary Fig. S3(a) revealed that the present electrodes possessed several hundred micrometers of pores and thus quite large electrochemical surface area. In the enlarged pictures for (b) NiMo cathode and (c) NiFe anode, the surface of Ni foam was uniformly covered by NiMo or NiFe species with different morphologies. The SEM images of Ni foam and NiFe were adopted from our recent study[1].

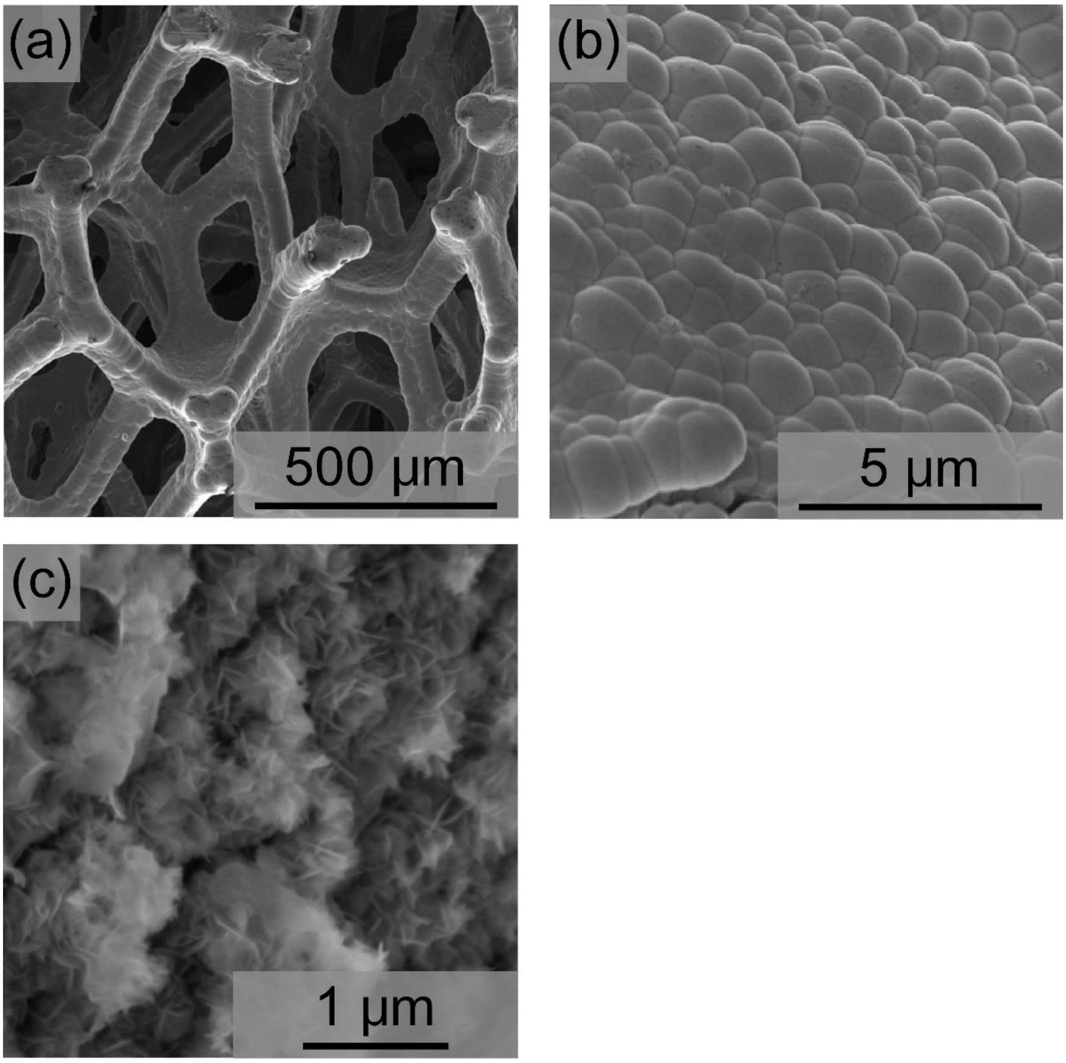


**Supplementary Figure S3.** SEM images for (a) bare Ni foam substrate, (b) NiMo deposited on the Ni foam, and (c) NiFe deposited on the Ni foam.

**Electric power of SPHELAR (3PVs and 4PVs) as a function of voltage**

The electric power generated by SPHELAR is plotted as a function of voltage in Supplementary Fig. S4. The same figure also compiles the power-voltage relationship of the electrocatalysts. Supplementary Fig. S4 clearly indicates that when the surface area of the electrocatalysts was adjusted, PVs can be operated at a plateau region or the power maximum point.

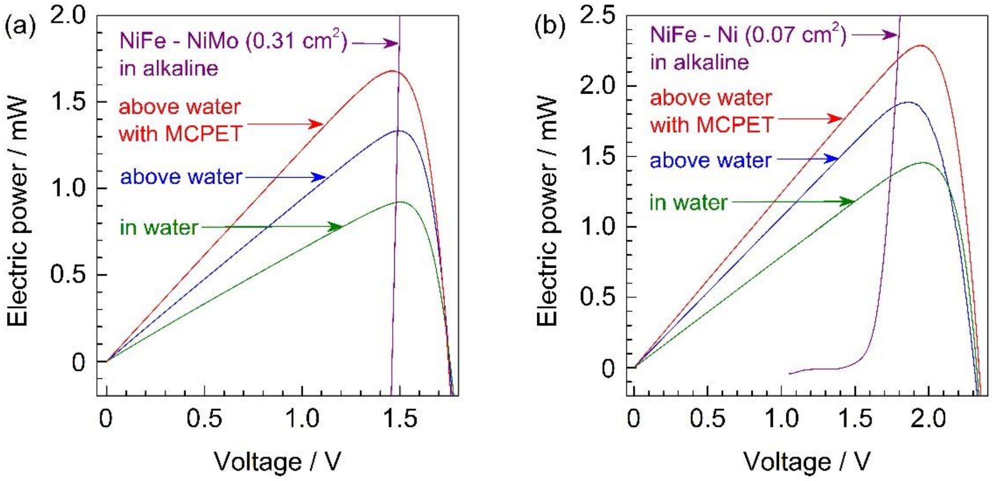


**Supplementary Figure S4.** The electric power for (a) 3PVs and (b) 4PVs as a function of voltage converted from corresponding *I*-*V* curves (Fig. 4). SPHELAR was fixed at approximately 1 cm depth of water, above water, and above water with MCPET illustrated in green, blue, and red lines, respectively. As representative choices of electrocatalysts, electric power for 0.31 $cm^2$ of NiFe – NiMo in alkaline solution for 3PVs and 0.07 $cm^2$ of NiFe – Ni in alkaline for 4PVs were also plotted. As a light source, solar simulator adjusted to AM1.5G was used.

**Summary of photovoltaic performances for SPHELAR with various configurations**

Supplementary Table S1 compiles the photovoltaic performances for SPHELAR (3PVs and 4PVs) with various configurations, where SPHELAR was fixed in water, above water, and above water with MCPET. Open-circuit voltage, $V_{OC}$, short-circuit current, $I_{SC}$, power maximum voltage, $V_{PM}$, and power maximum current, $I_{PM}$ (voltage and current generating maximum electric power, Supplementary Fig. S3) are summarized.

**Supplementary Table S1.** Summary of photovoltaic performances for SPHELAR with various configurations.

| Configurations | $V_{OC}$ / V | $I_{SC}$ / mA | $V_{PM}$ / V | $I_{PM}$ / mA |
|---|---|---|---|---|
| 3PVs in water | 1.76 | 0.689 | 1.50 | 0.613 |
| 3PVs above water | 1.75 | 0.971 | 1.50 | 0.893 |
| 3PVs above water with MCPET | 1.75 | 1.23 | 1.46 | 1.15 |
| 4PVs in water | 2.32 | 0.789 | 1.97 | 0.741 |
| 4PVs above water | 2.31 | 1.07 | 1.86 | 1.01 |
| 4PVs above water with MCPET | 2.34 | 1.24 | 1.94 | 1.18 |

**Summary of theoretical required geometric surface area for electrocatalysts**

The theoretical required geometric surface area for electrocatalysts discussed in the main manuscript (Fig. 5) were summarized in the following Supplementary Table S2.

**Supplementary Table S2.** Summary of theoretical required geometric surface area for electrocatalysts calculated in Fig. 5.

| Configurations | w/o MCPET | w/ MCPET |
|---|---|---|
| NiFe – NiMo in alkaline for 3PVs | 0.24 $cm^2$ | 0.31 $cm^2$ |
| NiFe – Ni in alkaline for 4PVs | 0.054 $cm^2$ | 0.070 $cm^2$ |
| NiCo – NiMo in near neutral for 4PVs | 0.62 $cm^2$ | 0.80 $cm^2$ |

**Calculation of the required minimum electrode surface area**

For the demonstration of overall water splitting with SPHELAR devices in the main manuscript, (NiFe, Pt/Ni) for 3PVs and (NiFe, Ni) for 4PVs in alkaline solution were used as the model cases for the combination of electrocatalysts. Notably, non-noble metal electrocatalysts, such as (NiCo, NiMo), can also be used, as discussed in Supplementary Fig. S2 and S4. The surface areas of the electrodes, e.g., (NiFe, Pt/Ni) for 3PVs and (NiFe, Ni) for 4PVs, were calculated in a similar manner as that in the main manuscript and are plotted in Supplementary Fig. S5. The required geometric surface areas were calculated as 0.34 $cm^2$, 0.45 $cm^2$, 0.14 $cm^2$, and 0.19 $cm^2$ for 3PVs without the reflector, 3PVs with MCPET, 4PVs without the reflector, and 4PVs with MCPET, respectively. These values were adopted in the actual experimental demonstration for overall water splitting by the SPHELAR devices. The small electrode geometric surface areas (less than 0.5 $cm^2$) likely contributed to the reduction of the catalyst amount and the prevention of shielding the absorber semiconductor from irradiated light.

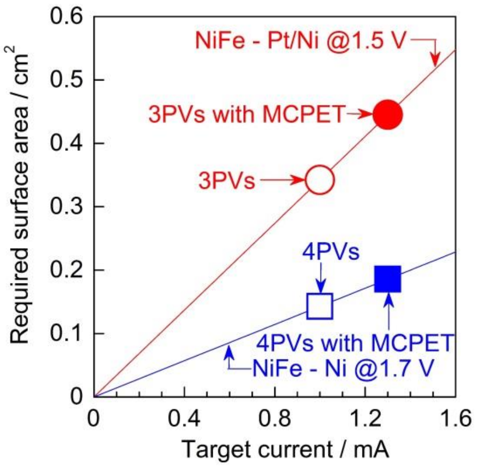


**Supplementary Figure S5.** Theoretically required surface area for electrodes (NiFe – Pt/Ni in alkaline solution for 3PVs, red line and NiFe – Ni in alkaline solution for 4PVs, blue line) as a function of target current at 1.5 V for 3PVs and 1.7 V for 4PVs calculated from current-voltage properties for SPHELARs (Fig. 4) and electrocatalysts (Supplementary Fig. S2). The actual geometric surface area that was applied to experimental section for 3PVs (○), 3PVs with MCPET (●), 4PVs (□), 4PVs with MCPET (■) were 0.34 $cm^2$, 0.45 $cm^2$, 0.14 $cm^2$, and 0.19 $cm^2$, respectively.

**Solution resistance measurement and prediction of *iR* drop under the experimental conditions for SPHELAR devices**

Solution resistances of the electrolyte (0.5 M KOH) were measured with impedance spectroscopy (100 kHz and 10 mV amplitude) in electrodes of various widths and heights and distances between the electrodes. As a model electrode, Ni foam was used for both the anode and the cathode with the same geometric size. In an electrochemical cell with a known cell constant, the resistivity of 0.5 M KOH was found to be 15.7 ohm cm. Supplementary Fig. S6 compiles the measured solution resistance as a function of the distance between the electrodes. Additionally, the resistance is plotted against *D/S*, where *D* is the distance between the electrodes and *S* is the surface area of the electrodes. The *D/S* term has the same unit as the cell constant [$M^{-1}$] in SI units. As a formal and convenient fitting, the cell constants of the present Ni foams were expressed in the formula: $K_{cell} = 0.44(D/S)^{0.27}$. The solution resistance and the corresponding *iR* drop in the actual SPHELAR configurations were predicted by the fitting and are plotted as a function of the distance between the electrodes in Supplementary Fig. 6. In these calculations, the following values were adopted:

(NiFe, Pt/Ni) for 3PVs with MCPET: $S = 0.45$ cm$^2$ and $D = 0.72$ cm,

(NiFe, Ni) for 4PVs with MCPET: $S = 0.19$ cm$^2$ and $D = 0.88$ cm.

Notably, *D* corresponds to the width of the body of the SPHELAR itself. The obtained *iR* drop was 10.3 mV and 13.8 mV for 3PVs and 4PVs, respectively. The small sizes of the device led to such a small *iR* drop in our system. Judging from the *I-V* properties for SPHELARs and electrocatalysts (Fig. 4), these *iR* drops were considered to be quite small and negligible for PV performance (less than 1% of the overall voltage).

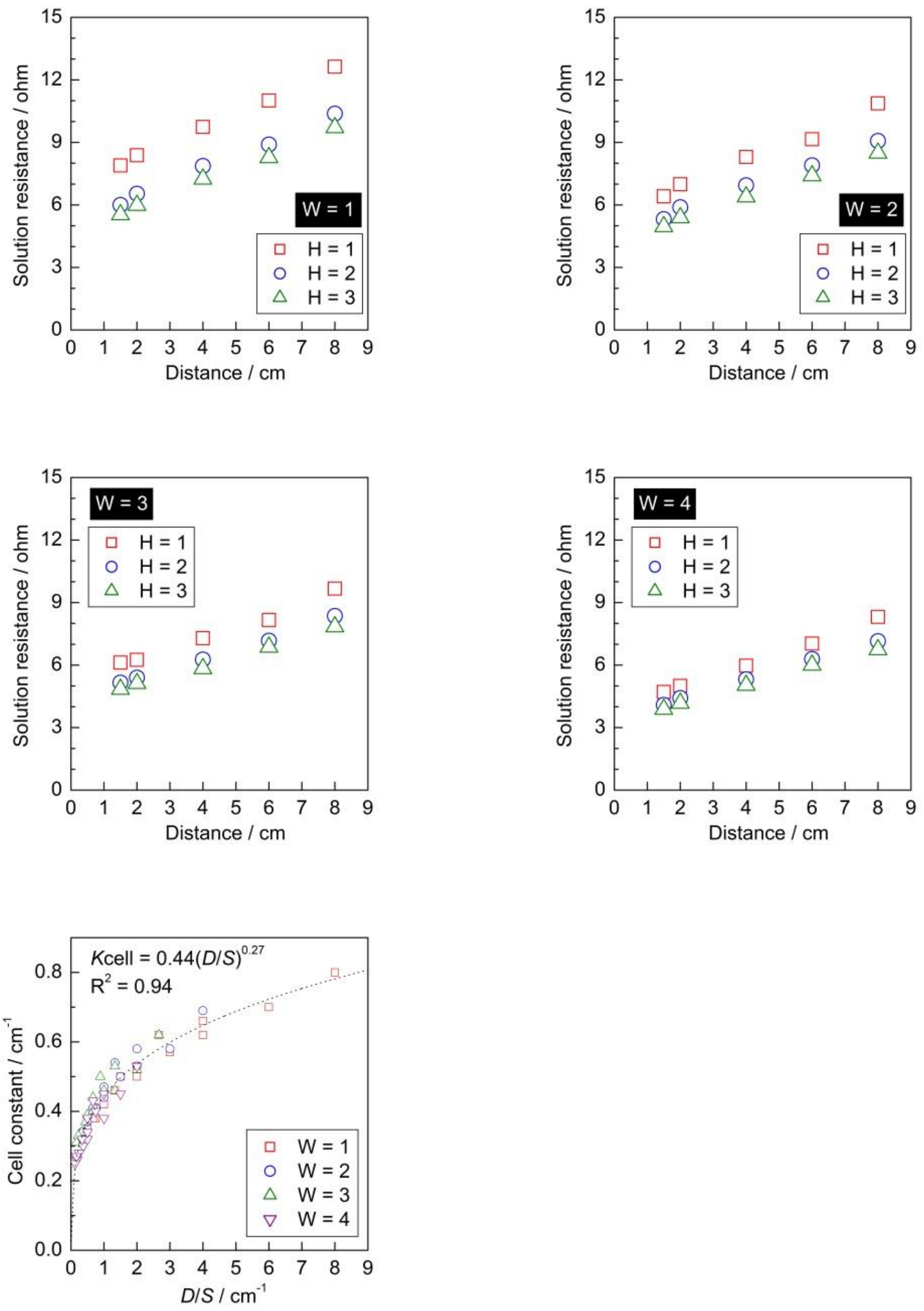


**Supplementary Figure S6.** Solution resistance for 0.5 M KOH as a function of distance between anode and cathode, that were also converted to cell constant vs. *D*/*S*, where *D* and *S* indicate distance and geometric surface area for electrodes, respectively. Ni foam was used for both anode and cathode as a representative electrode. The electrochemical impedance spectroscopy (100 kHz and 10 mV amplitude) were performed in various configurations: various width (1 – 4 cm), height (1 – 3 cm) and distances (1.5 – 8 cm) of Ni electrodes.

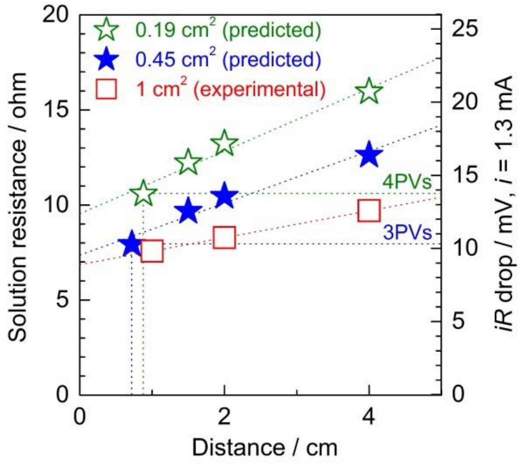


**Supplementary Figure S7.** Expected solution resistance and corresponding value of *iR* drop as a function of distance between electrodes calculated from the results of the electrochemical impedance spectroscopy (Supplementary Fig. S6).

**Demonstrations of overall water splitting by SPHELAR devices**

As noted in Section 3-3 of the main manuscript, the overall water splitting with our SPHELAR devices, composed of (NiFe, Pt/Ni) for 3PVs and (NiFe, Ni) for 4PVs in 0.5 M KOH, was also demonstrated in a batch reactor. The electrode surface areas were 0.34 $cm^2$ for 3PVs without MCPET and 0.14 $cm^2$ for 4PVS without MCPET, as discussed in Supplementary Fig. S5. In this section, various SPHELAR configurations, where the 3PV or 4PV body was fixed in the electrolyte and above the electrolyte without the MCPET reflector, were evaluated. The overall water splitting was performed in a Pyrex top-irradiation cell connected to a recirculating batch reactor at room temperature, where evolved gases were analyzed by gas chromatography (Shimadzu Co. Ltd., GC-8A), with the instrument equipped with a TCD detector and a MS-5A column using Ar as a carrier gas, as described in the experimental section. In all of the time courses plotted in Supplementary Fig. S8, the evolved hydrogen and oxygen amounts monotonically increased during light irradiation for 2.0 h. After the light was turned off, no further formation or consumption of hydrogen and oxygen was observed, thus confirming that the gas productions were induced by harvesting light and that no apparent reverse reaction of products forming water in the dark took place. The ratio of evolved hydrogen over oxygen was approximately 2, which agreed with the stoichiometry of the water splitting reaction. The hydrogen and oxygen production rate in Supplementary Fig. S8 agreed with the expected photoelectric and electric currents (see Fig. 4), revealing that an approximately 100% of Faradic efficiency was attained. These observations were all consistent with the flow reactor discussed in Figs. 6 and 7 in the main text.

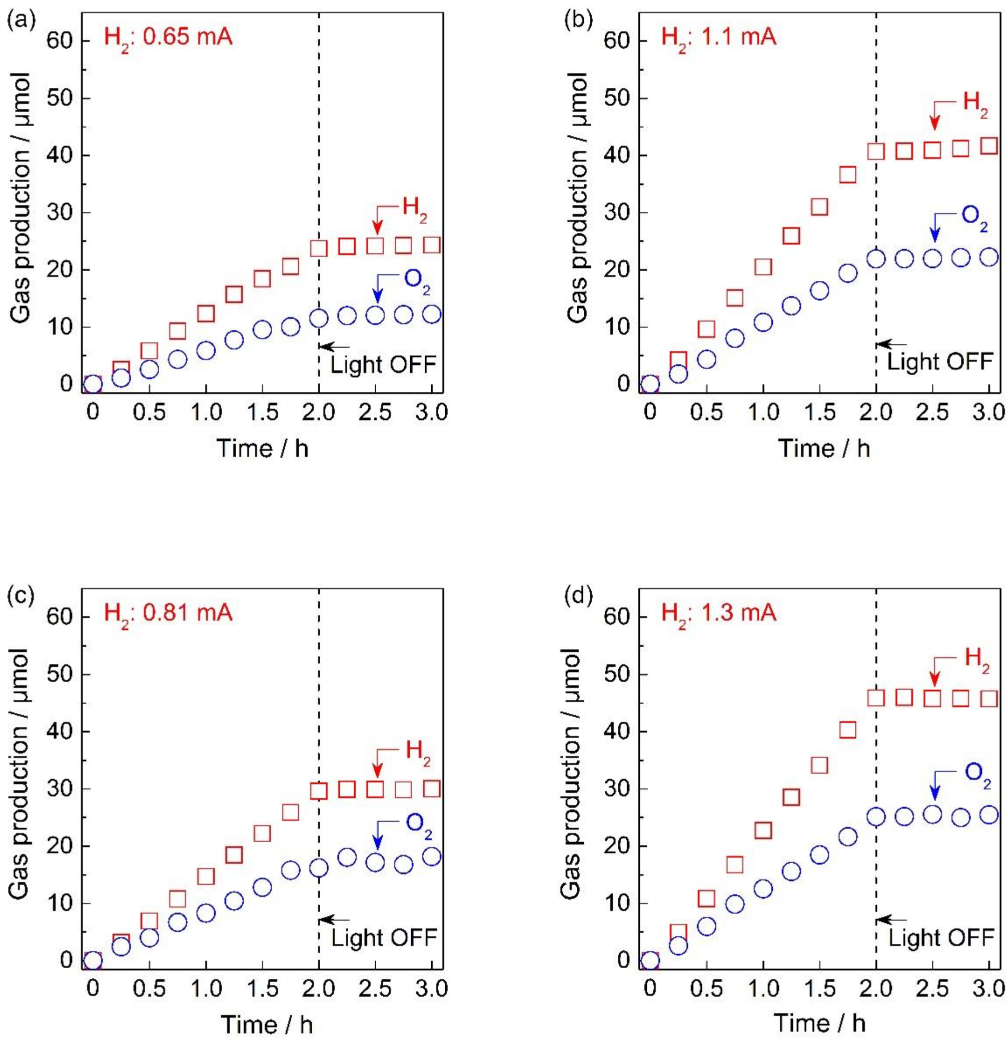


**Supplementary Figure S8.** Time courses of overall water splitting into $H_2$ (□) and $O_2$ (○) measured in batch reactor by (a) 3PVs fixed in 0.5 M KOH aqueous solution as an electrolyte, (b) 3PVs fixed above the electrolyte, (c) 4PVs fixed in the electrolyte, and (d) 4PVs fixed above the electrolyte under irradiation of solar simulator (AM1.5G) as a light source. As the electrocatalysts, combination of NiFe-LDH (anode) and Pt/Ni (cathode) and that of NiFe(anode) and Ni foam (cathode) were applied for 3PVs and 4PVs, respectively. The reactions were measured in a Pyrex top-irradiation cell connected to the recirculating bath reactor at room temperature.

**Movie of an experimental demonstration for overall water splitting by a stand-alone SPHELAR device consisting of 3PVs with MCPET and (NiFe, Pt/Ni) as electrocatalysts**

The overall water splitting by a stand-alone SPHELAR device demonstrated in the recirculating batch reactor (as shown in Supplementary Fig. S8) is shown in Supplementary Movie S1. The body of the 3PVs was supported by the electrodes and settled above the electrolyte. MCPET as a reflector was located under the SPHELAR (3PVs) with the same geometric size as the body of the SPHELAR. Spontaneous gas evolution from both the anode (NiFe) and the cathode (Pt/Ni) can be seen in the movie.

**Supplementary Movie S1.** Movie of overall water splitting by the stand-alone SPHELAR device consisting of 3PVs with MCPET and NiFe – Pt/Ni as a reflector and electrocatalysts, respectively.

**Light intensity of the solar simulator used as a light source**

Supplementary Fig. S9 illustrates the spectra of the solar simulator used in the present study. As a reference, the AM1.5G spectrum is also depicted. The spectrum of the solar simulator matched well with that of the AM1.5G.

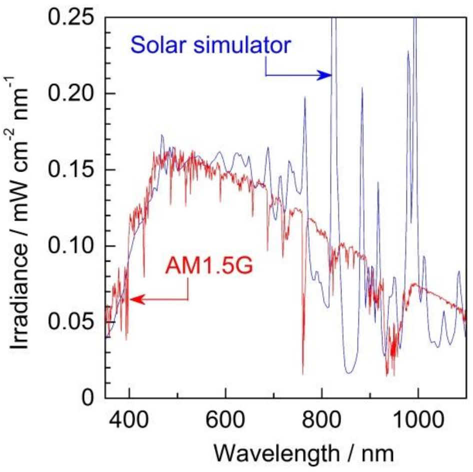


**Supplementary Figure S9.** Spectra of the solar simulator utilized as a light source in the present study (blue line) and AM1.5G (red line)

**Summary of achieved STH efficiencies**

Supplementary Table S3 compiles the photocurrents in various module configurations, calculated from the evolved $H_2$ rate. Also, the corresponding STH efficiency is summarized.

**Supplementary Table S3.** Summary of $H_2$ generation rate (mA) and STH efficiency for overall water splitting by stand-alone SPHELAR devices in various configurations. Projected surface area of 0.20 cm$^2$ for 3PVs and 0.26 cm$^2$ for 4PVs were applied for STH calculation.

| | **$H_2$ / mA** | **STH / %** |
|---|---|---|
| 3PVs in | 0.79 | 4.9 |
| 3PVs above | 1.0 | 6.1 |
| 3PVs/MCPET above | 1.2 | 7.4 |
| 4PVs in | 0.82 | 3.9 |
| 4PVs above | 1.0 | 4.8 |
| 4PVs/MCPET above | 1.3 | 6.4 |

Reference

1. Nurlaela, E., Shinagawa, T., Qureshi, M., Dhawale, D. S. & Takanabe, K. Temperature Dependence of Electrocatalytic and Photocatalytic Oxygen Evolution Reaction Rates Using NiFe Oxide. *ACS Catal.* **6**, 1713−1722 (2016).
